# Comparison of the physiological responses and time-motion characteristics during football small-sided games: effect of pressure on the ball

**DOI:** 10.3389/fphys.2023.1167624

**Published:** 2023-05-19

**Authors:** Xiaohu Chen, Rui Zheng, Bo Xiong, Xiaoling Huang, Bingnan Gong

**Affiliations:** ^1^ Sports College, Southwest University, Chongqing, China; ^2^ Sport Department, Chongqing Liangjiang Secondary School, Chongqing, China; ^3^ Institute of Physical Education and Training, Capital University of Physical Education and Sports, Beijing, China

**Keywords:** pressing, heart rate, running, games, football (soccer)

## Abstract

**Introduction:** This study aimed to compare the effects of pressure on the ball on physiological responses and time-motion characteristics during football small-sided games between elite youth male players.

**Methods:** 56 elite male youth football players (age: 15.43 ± 0.52 years) performed a 2+GK vs. 2+GK game on a 30 m × 15 m pitch area with two playing conditions: 1) free play (FP), the player has no limitation to play; 2) pressure on the ball (PB), the player has directly and aggressively closed down space (located within 1.5 m) between themselves and the opposition player with the ball and can compete for possession. The percentage of time spent in different maximum heart rate (HRmax) zones, mean heart rate, blood lactate acid concentration, total distance covered, distance covered in three speed zones (sprint, high speed, and moderate speed), number of high speed runs, number of sprint runs, top speed, number of direction changes, and ball recovery time were monitored.

**Results:** We found very significantly higher number of high speed runs (*p* < 0.001; ES = 1.154), number of direction changes (*p* < 0.001; ES = 2.347), ball recovery time (*p* < 0.001; ES = 3.529), percentage of time spent in 90%–100% HRmax (*p* < 0.001; ES = 3.033), mean heart rate (*p* < 0.001; ES = 1.940), blood lactate acid concentration (*p* < 0.001; ES = 2.245) and significantly higher high speed running distance covered (*p* = 0.004; ES = 0.520) in the PB condition. Conversely, the FP condition showed very significantly higher moderate speed running distance covered (*p* < 0.001; ES = 1.814) and significantly higher percentage of time spent in 80%–90% HRmax (*p* = 0.012; ES = 0.440). No significant differences were revealed on sprint running distance covered (*p* = 0.407; ES = 0.140), number of sprint runs (*p* = 0.103; ES = 0.279), top speed (*p* = 0.130; ES = 0.258) and percentage of time spent in 60%–70% HRmax (*p* = 0.106; ES = 0.276), 70%–80% HRmax (*p* = 0.358; ES = 0.155).

**Discussion:** We found that pressure on the ball had a substantial impact on the intensity of training, as evidenced by a significantly increased high speed running performance, number of directional changes, percentage of time spent at 90%–100% of maximum heart rate, mean heart rate, and blood lactate acid concentration. Additionally, ball recovery time decreased significantly.

## Introduction

Small-sided games (SSGs) are often described as a small version of the real game (FIFA, 2014). It is commonly used as a training modality that includes a reduced pitch area, adjusted game rules, and a smaller number of players than a real game (Hill-haas et al., 2011). Compared to other training methods, it not only provides training with realistic game situations (Gregson and Drust, 2000; Owen, 2003), but also ignites players’ enthusiasm to participate in training. By adjusting different factors, coaches can also enable players to practice different types of movement in a real game situation (Aroso et al., 2004) as well as different types of technical under pressure (Gabbett et al., 2009). Moreover, it trains players to make various tactical decisions when they are physically exhausted (Castelao et al., 2014) and increases their mental toughness (Diogo et al., 2017). In order to reproduce the physical, technical and tactical requirements of real match play, coaches often use SSGs in their training programs.

The evolution of GPS and heart rate monitor devices (Edwards, 1993; Barbero-Álvarez et al., 2010; Coutts and Duffield, 2010) and the observational tactical instrument tools (Gonzalez- Villora et al., 2015) help coaches and scientists to study the physiological, biomechanical, technical and specific tactical characteristics of different SSGs has increased exponentially in the last years. Most of the studies were focused on the following aspects:

1) The number of players. Studies showed that lower number of players (1v1 to 4v4) significantly increase the physiological demand compared to medium-sided games (5v5–8v8) and large-sided games (>9v9). Heart rate, blood lactate acid, and rating of perceived exertion decreased as the number of players increased (Aroso et al., 2004; Owen et al., 2004; Williams and Owen, 2007; Katis and Kellis, 2009; Dellal et al., 2011; Aguiar et al., 2013). Other studies found that the total distance covered increased with an increasing number of players (Dellal et al., 2011; Clemente et al., 2014a), but the number of accelerations, decelerations, changes of direction, and sprints decreased (Hill-Haas et al., 2008; Aguiar et al., 2012; Clemente et al., 2017; Lacome et al., 2018). There is an increase in the number of technical actions performed per player with smaller-sided games (Almeida et al., 2013; Clemente et al., 2014b; Joo et al., 2016), but more tactical decisions are made in larger-sided games (Katis and Kellis, 2009; Teoldo et al., 2010; Owen et al., 2014; Joo et al., 2016).
2) Pitch size. A number of studies have shown that physical loads (heart rate, blood lactate and RPE) increase when the pitch size per player increases (Aslan, 2013; Köklü et al., 2013; Hodgson et al., 2014; Pantelic et al., 2019). On the other hand, some studies reveal that the larger pitch area leads to an increase intotal running distance, high-speed running, as well as number of accelerations and decelerations (Casamichana and Castellano, 2010; Hodgson et al., 2014). Meanwhile, the number of movements that player off the ball (Almeida et al., 2013; Joo et al., 2016) and tactical behaviors (Castelao et al., 2014; Silva et al., 2014) were increased in larger pitch area, while the number of actions that player perform with the ball tends to decrease (Katis and Kellis, 2009; Da Silva et al., 2011; Almeida et al., 2013).
3) The game rules. Different game rule modifications may lead to different responses during SSGs. Research has shown that rule changes also have a major impact on the physiological, technical and tactical performance, such as different types of marking (man-to-man, double marking, and zonal) (Casamichana et al., 2015; Clemente et al., 2016), number of ball touches (Clemente et al., 2014a; Sousa et al., 2019), scoring method (Clemente et al., 2014b), goal format (Craig et al., 2016; Coutinho et al., 2018), goalkeepers (Köklü et al., 2015) and floater player (Mallo and Navarro, 2008).

In recent years, pressure on the ball, popularised by coaches such as Jürgen Klopp and Pep Guardiola, has become a highly influential defensive tactical action within the game. The importance of defense in football has been highlighted in various studies (Bojan et al., 2019; Lepschy et al., 2021; Low et al., 2021). Defensive tactics like the high-intensity pressing and counter pressing (Nassis et al., 2020; Harper et al., 2021) are considered the most important tactical concepts in how the defensive game will be played in the future. Pressing is all about applying pressure of the players with the ball so that they no longer have the freedom to decide what to do with it and influencing their decision making (Link et al., 2016; Andrienko et al., 2017), which is also very important for the team to have back the ball (Casal-Sanjurjo et al., 2020) and as a starting point for counter attacking (Herold et al., 2022). However, to the best of our knowledge, no studies have reported the effects of the pressure on the ball on physiological responses and time-motion characteristics during football SSGs. Therefore, this leads to the hypothesis that restricting this factor has an impact on training effects, and that it influences the behavior of players during training. The aim of this study was to compare the effects of free play and pressure on the ball on physiological responses and time-motion characteristics during football SSGs.

## Methods

### Experimental approach to the problem

For the SSGs, previous studies have shown that more intensity and higher frequency of running (Owen et al., 2004; Williams and Owen, 2007; Koklu et al., 2015) are generally found in a smaller number of players, resulting in very high levels of effort (Clemente et al., 2014a). In addition, the use of goalkeepers can stimulate the enthusiasm of players to participate in training (Koklu et al., 2015). Moreover, a larger goal also encourages players to keep moving so they can shoot from different angles (Coutinho et al., 2018), leading to more transitions of the ball possession. Based on the pitch size, the individual area for each player (the relative pitch area per player) is determined (Pantelic et al., 2019). Studies have shown that the greater the area per player, the greater the physical load (Aroso et al., 2004). To provide a frame of reference, the average area per player ranged from 83.2 to 117 m^2^ in the 2018 Russia World Cup (FIFA, 2018). The shape of the pitch is also considered because a rectangular shape is more likely to increase the penetrations rather than going wide (Coutinho et al., 2018).

**FIGURE 1:**
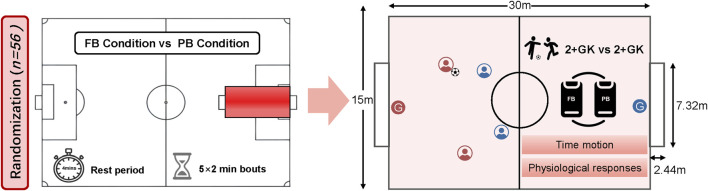
The field design of small-sided games.

Based on the above factors, the SSGs in this study consist of 2+ GK vs. 2+ GK sessions on a 30 × 15 m pitch area (length × width, 117 m^2^ relative pitch area per player) with two standard goals (7.32 × 2.44 m). Players score a goal by shooting into the normal goals; the goalkeeper restarts the game after the ball goes out of play. Several balls are placed beside the two goals to ensure that the game can be restarted as quickly as possible (See Figure 1). The offside rule was not enforced in either condition, while no coach feedback or encouragement was allowed during the SSGs (Batista et al., 2019). The SSGs includes 5 × 2-min bouts interspersed with a 4-min passive recovery period.

**FIGURE 2:**
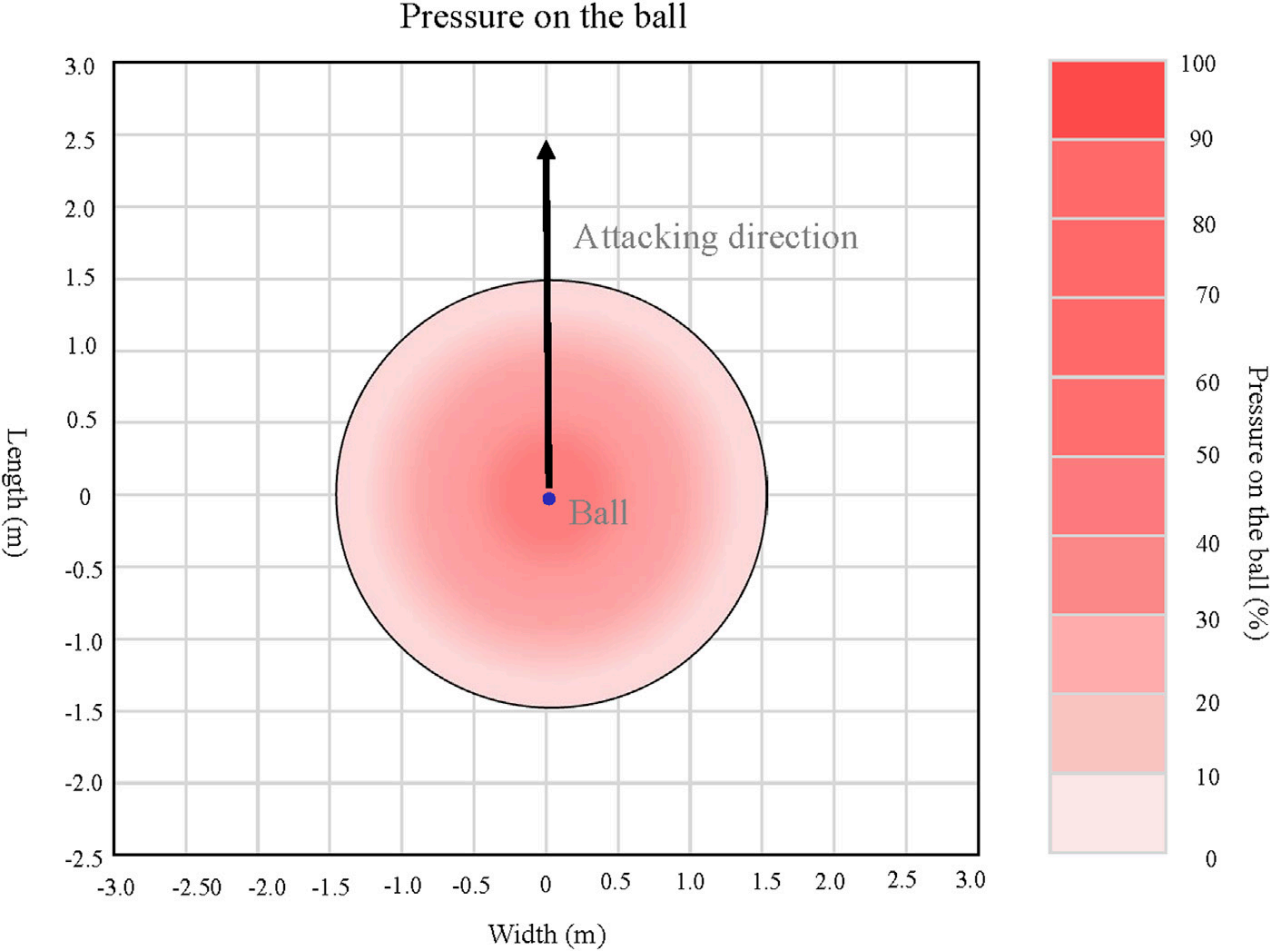
Pressure on the ball.

The experiment was based on repeated SSGs with two different conditions: FP = Free play condition, PB = Pressure on the ball condition. The FP condition was the experiment under the free play (FP) condition in which player has no limitation to play, it was carried out 5 times a week for 3 weeks, totally 15 times. In the PB conditions, the experiment was conducted the pressure on the ball (PB) condition in which player has directly and aggressively closed down space (located within 1.5 m) between themselves and the opposition player with the ball and can compete for possession (See Figure 2). It also carried out 5 times a week for 3 weeks, for a total of 15 times. All the participants were randomly assigned to the experiment. In addition, the inter-trial interval was randomized between 3 weeks to prevent any potential physiological adaptation that may have influenced the results.

### Subjects

56 elite male soccer players consisting of 48 field players and 8 goalkeepers (age:15.43 ± 0.52; height: 176 ± 5.4 cm; weight: 61 ± 6.1; years of training: 7 ± 1.3; mean maximum heart rate: 208 ± 9.3 beats/min). All players were selected from four professional soccer clubs (12 outfield players and 2 goalkeepers for each) by their team’s head coach based on their level of skill (technical, tactical and fitness) and without consideration of their on-field positions. All of them participated in the National Youth Super League. They had been training with the club for at least 5 years, for approximately 40 weeks per year, with each week consisting of 6–8 training sessions (each session lasts 90–120 min). Although goalkeepers participated in this study, the relevant data was not recorded owing to the limited activity at their position. All players were informed of the research design and the potential benefits and risks, while written consent was obtained prior to participation. Ethical approval was granted by the institutional human research ethics committee.

### Procedures

The experiment was conducted during the pre-match preparation period of each team. The SSGs were played on a natural grass surface at the same time of day (16:00–17:30) under sunny conditions (19.5°C ± 1.4°C) to avoid the effects of circadian rhythms on the results. Prior to each session, participants were instructed to refrain from caffeine (4 h), strenuous exercise and alcohol consumption (48 h), and to arrive in a fully rested and hydrated state. Participants were also provided with a 24 h food diary and asked to record their diet for the 24 h before each trial.

Each session started with a standardized 20-min warm-up that included jogging, stretching, quick feet, and a ball possession game (6-a-side without goals). Goalkeepers warmed up individually with the goalkeeping coach, and warm-up activities included jogging, stretching, quick feet, and catching. Then, the players were randomly assigned into two balanced teams and instructed to perform the relevant tasks. For each session, the players remained in the same team and played with the same pairs in 2v2.

## Data collection

### Physiological responses data

Heart rate was recorded at 5-s intervals during each SSG via short-range radio telemetry (Polar Team Sport System; Polar Electro Oy, Kempele, Finland). The heart rate monitors were worn during the 20-m shuttle run assessments (Bangsbo, 1994) to determine the player’s HRmax. Training intensity was expressed as a percentage of HRmax and classified as the percentage of time spent in the following zones of intensity (Eniseler, 2005): Zone 1 (60%–70% HRmax), Zone 2 (70%–80% HRmax), Zone 3 (80%–90% HRmax), and Zone 4 (90%–100% HRmax). The mean heart rate under both conditions was determined to indicate the overall intensity. Blood lactate samples were taken 4 min after the end of each bout from each player in both conditions, while blood samples were taken from their ear lobes and immediately analyzed using a Lactate Pro (LT-1710; Arkray, Kyoto, Japan) analyzer that had been previously validated (Hill-Haas et al., 2008).

### Time-motion characteristics data

Movement patterns during the games were measured using a portable global positioning satellite system (GPS) (GP Sports SPI Elite System, Canberra, Australia), in which the distance travelled was recorded at 5 Hz. This technology has been previously determined to be reliable for monitoring high-intensity and sprinting activities in soccer (Coutts and Duffield, 2010), especially during exercises that include repeated sprints (Barbero-Álvarez et al., 2010). For data analysis, three running intensity zones were selected (FIFA, 2018): Zone 1 (moderate speed running, 15–20 km/h), Zone 2 (high speed running, 20–25 km/h), Zone 3 (sprint running, ≥25 km/h). The software calculated the total distance, the distance covered in the three intensity running zones, the number of high speed runs and sprint runs, top speed, the number of direction changes, and ball recovery time were monitored.

### Statistical analysis

Data are presented as mean ± standard deviation (M±SD). Before using parametric statistical analyses procedures, the normality of the data was verified by the Shapiro-Wilks test. A paired sample *t*-test was used to compare the differences in physiological responses and time-motion characteristics under the FP and PB conditions. Differences were considered significant at *p* < 0.05. Cohen’s effect sizes were also calculated to describe any trends apparent in the data. The scale identified an effect size of 0.2 as representing a small effect, 0.5 a moderate effect, and 0.8 or greater as a large effect (Rhea, 2004; Tomczak and Tomczak, 2014). Statistical analysis was performed using SPSS software (version 26.0; SPSS Inc., Chicago, IL, United States).

## Results

Table 1 presents the results of the comparison between the FP and PB conditions for physiological responses. Compared with the FP condition, the PB condition notably revealed a significantly increase in the percentage of time spent in 90%–100% HRmax (*p* < 0.001; ES = 3.033), mean heart rate (*p* < 0.001; ES = 1.940), blood lactate acid (*p* < 0.001; ES = 2.245). However, the percentage of time spent in 80%–90% HRmax (*p* = 0.012; ES = 0.440) was significantly decreased. There was no significant difference in the percentage of time spent in 60%–70% HRmax (*p* = 0.106; ES = 0.276) and 70%–80% HRmax (*p* = 0.358; ES = 0.155).

**TABLE 1:** Comparison of physiological responses in different conditions.

Physiological variables	FP condition	PB condition	t	*p*	Cohen’ d
60%–70% HRmax (%)	13.25 ± 1.68	12.78 ± 1.05	1.66	0.106	0.276
70%–80% HRmax (%)	25.57 ± 2.87	26.11 ± 1.74	−0.93	0.358	0.155
80%–90% HRmax (%)	39.83 ± 3.87	37.43 ± 3.25	2.64	0.012*	0.440
90%–100% HRmax (%)	21.36 ± 3.19	23.82 ± 3.10	−18.19	<0.001**	3.033
Mean heart rate (beats/min)	179.24 ± 2.86	182.01 ± 2.80	−11.64	<0.001**	1.940
blood lactate acid (mmol/L)	8.83 ± 1.12	10.91 ± 1.17	−13.47	<0.001**	2.245

Notes: FP, free play condition; PB, Pressure on the ball condition. Legends: * = *p* < 0.05, ** = *p* < 0.001.

For the movement pattern, Table 2 presents the time-motion analysis results of the FP and PB conditions. In the PB condition, there was a very significant increase in the number of high speed runs (*p* < 0.001; ES = 1.154), number of direction changes (*p* < 0.001; ES = 2.347) and ball recovery time (*p* < 0.001; ES = 3.529), and significant increase in the high speed running (*p* = 0.004; ES = 0.520), but the moderate speed running (*p* < 0.001; ES = 1.1814) was significantly decreased. However, there were no significant differences between sprint running (*p* = 0.407; ES = 0.140), total distance (*p* = 0.092; ES = 0.289), number of sprint runs (*p* = 0.103; ES = 0.279) and top speed (*p* = 0.130; ES = 0.258) between conditions.

**TABLE 2:** Comparison of time motion analysis in different conditions.

Movement variables	FP condition	PB condition	t	*p*	Cohen’ d
Moderate speed running (m/min)	33.63 ± 4.50	22.36 ± 3.15	10.88	<0.001**	1.814
High speed running (m/min)	10.63 ± 2.88	12.46 ± 2.22	−3.11	0.004*	0.520
Sprint running (m/min)	6.24 ± 1.90	6.50 ± 0.72	−0.83	0.407	0.140
Total distance (m/min)	132.56 ± 9.12	136.22 ± 6.74	−1.73	0.092	0.289
Numbers of high speed runs (times/min)	5.19 ± 2.25	8.80 ± 1.80	−6.92	<0.001**	1.154
Numbers of sprint runs (times/min)	1.27 ± 0.51	1.50 ± 0.77	−1.67	0.103	0.279
Top speed (m/s)	7.44 ± 0.20	7.48 ± 0.24	−1.55	0.130	0.258
Numbers of direction changes (times/min)	5.25 ± 1.50	12.13 ± 2.23	−14.08	<0.001**	2.347
Ball recovery time(s)	6.36 ± 0.68	3.11 ± 0.55	14.97	<0.001**	3.529

Notes: FP, free play condition; PB, Pressure on the ball condition. Legends: * = *p* < 0.05, ** = *p* < 0.001.

## Discussion

In general, restricting the pressure on the ball had a significant effect on training intensity, we found a significantly higher number of high speed runs, high speed running distance covered, number of direction changes, percentage of time spent at 90%–100% HRmax, mean heart rate, blood lactate acid concentration, as well as decreased ball recovery time. However, its effect on sprinting movement was not readily apparent.

The mean heart rate of the players in FP was 86% HRmax, and most of the time was spent in the zone with 80%–90% HRmax, accounting for 39.83% of the total time. This is similar to the previous researches by Halouani et al. (2017) and Dellal et al. (2011), where the mean heart rate was 84.2% HRmax and 85.1% HRmax respectively in 2V2 SSGs. The mean heart rate of players in the experiment met the requirements of a soccer match, as the average work intensity measured by HRmax in previous matches revealed a profile of activity close to the anaerobic threshold (80%–90% HRmax) (FIFA, 2014). After the restriction of pressure on the ball, the mean heart rate increased significantly to 88% HRmax, which is higher than the evidence from a U18 national team recorded during the match (FIFA, 2018). The reason is that when the pressure on the ball is restricted, the defender nearest the ball will close down to the ball more quickly to pressure the opponent, so the player in possession of the ball does not have much time to make a decision, which results in more transfer of ball possession. Meanwhile, the player who gain possession of the ball are also more likely to launch counter-attacks, which also increase the intensity of the training. The finding was consistent with previous studies showing that players using man-to-man defense strategies in SSGs tend to display improved training intensity (Aroso et al., 2004; Casamichana and Castellano, 2010).

Establishing the training intensity of players cannot solely rely on measuring their heart rate responses. There was also a very significant increase in blood lactate acid in PB condition, that is, higher than the lactate threshold recorded in official matches (Bangsbo, 1994). The result was consistent with the findings of Gerisch et al. (1988), Hill-Haas et al. (2011); Halouani et al. (2017), who showed that players could reach peak blood lactate acid in 1v1 or 2v2 format. In addition to the aforementioned increase in transitions, the number of finishes/shots will also increase significantly, which may also lead to an increase in lactate acid concentration. Conversely, if there is no pressure on the ball restriction, players who are tired will choose to protect the ball or pass the ball, which will not stimulate higher lactate levels. Similarly, pressing immediately after losing the ball will also stimulate higher levels of blood lactate acid production (Aroso et al., 2004; Cihan, 2015). The advantage of counter-pressing allows player to restart an attack without having to go through a defending phase and to win the ball back in areas of the pitch close to the goal. Therefore, constraint the pressure on the ball has a very important effect on the tactical production of high-pressing and counter-pressing, which can help players to maintain a high intensity performance in a relatively short period of time (Bangsbo, 1994; Eniseler, 2005).

In terms of movement, there was no significant difference in total distance covered during FP and PB conditions. However, the high-speed running especially number of high-speed runs were significantly improved, while the distance of moderate-speed running was significantly decreased after the transition time was restricted. In a similar SSG study, it was reported that double man pressure has significantly increased high-intensity running (Cihan, 2015; Casamichana et al., 2015). These findings could be explained by: 1) the pressure on the ball stimulated the defenders close down the opponent more quickly both in pressing and counter-pressing scenarios, while the attacking players are also forced to perform extra movement in an attempt to lose their pressure and create space (Cihan, 2015), the result showed that the ball recovery time was very significantly decrease from 6.36 to 3.11 s; 2) Pressing towards the ball inevitably means leaving space elsewhere on the pitch, players are required to perform a higher number of direction changes and high speed runs to recover the ball. It can be confirmed by the very significant increase in number of direction changes, which was from 5.25 to 12.13 times/min. When constraint the pressure on the ball, players have to directly and aggressively close down space between themselves and opponents with the ball, but the more quickly close to the ball the more risk will leave behind (Harper et al., 2021). Therefore, it is vital that when the press occurs, players know the trigger points and the details of the press; 3) Pressure on the ball also stimulated the player’s anticipation to move forward, which encouraged players to make a greater number of high speed runs. In PB situation, player attempted to closed down the space between themselves and an opposition player even the opposition player does not have the ball. Players are trying to reading body positions to anticipate where the ball is going and move out to meet it immediately, providing no respite for the opposition (Nassis et al., 2020). This is also a vital element to a good press. In addition, we also found that there is not one moment where one of the players are not completely focused in PB situation. It is a characteristic of the modern game–it demands full concentration for 90 min, especially if you want to apply the quality of that press.

However, one of the indicators was in line with experimental expectations, namely, that there was no significant difference in top speed, sprint running distance and number of sprint runs between conditions. This can be partially explained by two aspects. On the one hand, the likelihood of a pitch size effect, as we know it is difficult for the players to reach the top speed within limited area especially with short length. A study conducted in U17 players revealed that increased sprinting performance were performed in the two bigger area dimensions (175 and 273 m^2^ per player) (Casamichana and Castellano, 2010). Similar observation has also been made with the bigger area dimensions (200 m^2^ per player) led to an increased number of accelerations and decelerations (Hodgson et al., 2014). On the other hand, the number of players has also impacted the sprinting performance. The previous study showed that the top speed reached was greater during 9 vs. 9 SSGs than in 3 vs. 3 games (Casamichana et al., 2015). This is consistent with previous research that had found higher sprint duration in games involving more players and places (Hill-Haas et al., 2009; Brandes et al., 2011).

## Limitations

However, some limitations of our work should be acknowledged. Although previous research has demonstrated that the Rating of Perceived Exertion can provide supporting evidence for post-training physiological responses, the present study was unable to obtain relevant data samples. Therefore, future research should include such measures. Owing to the lack of data on the players’ focus and anticipation metrics during the experiment, the present study was unable to determine their psychological performance feedback. In addition, due to the limitations of technical equipment, many technical and tactical metrics were not collected in this study.

## Practical applications

As a key factor in the development of modern football, coaches can restrict the pressure on the ball as a control factor in training, which can effectively improve the intensity of training to even surpass that of a real match. The result observed from this study just reflected the youth elite players’ abilities, coaches need to adjust the distance location when the defender close down space between themself and the player in possession of the ball according to the age, skill level, tactical level, and training purpose of their players. While simultaneously monitoring the players in real-time through wearable devices to achieve different training outcomes. From the perspective of periodization of training, the two conditions of SSGs in the experiment can develop the special anaerobic endurance of athletes during pre-season training; during the season, it can better stimulate players’ high-intensity load and mental concentration by PB condition.

## Data Availability

The original contributions presented in the study are included in the article/Supplementary Material, further inquiries can be directed to the corresponding author.
